# A preliminary assessment of the LMA protector™ in non-paralysed patients

**DOI:** 10.1186/s12871-017-0323-5

**Published:** 2017-02-20

**Authors:** Ban Leong Sng, Farida Binte Ithnin, Deepak Mathur, Eileen Lew, Nian-Lin Reena Han, Alex Tiong-Heng Sia

**Affiliations:** 1 Department of Women’s Anaesthesia, KK Women’s and Children’s Hospital, 100 Bukit Timah Road, Singapore, Singapore; 2 Duke-NUS Medical School, 8 College Road, Singapore, Singapore; 3 Division of Clinical Support Services, KK Women’s and Children’s Hospital, 100 Bukit Timah Road, Singapore, Singapore

**Keywords:** Airway management, Laryngeal mask, Anaesthesia

## Abstract

**Background:**

The LMA Protector™ is the latest CE marked single use supraglottic airway device. This airway device provides access and functional separation of the respiratory and digestive tracts. There are two ports (male, female ports) to provide suction in the laryngeal region and insertion of the gastric tube. The aim of our study is to assess the ease of use, airway quality, device positioning, airway leak and complications associated with initial clinical experience in LMA Protector™ usage.

**Methods:**

This is an initial investigation of LMA Protector™ airway device. We conducted a preliminary assessment in the anaesthetised women who underwent minor gynaecological procedures with spontaneous ventilation in order to evaluate the performance of the airway device.

**Results:**

Insertion was successful on first and second attempts in 23 (88.5%) and 3 (11.5%) respectively. Median [IQR (range)] insertion time was 19 [17-21(14-58)] seconds. Airway leak pressure was 25.5 [23-29(21-30] cmH_2_O. On fibreoptic examination via the device, vocal cords were visible in all 26 patients. There were no alternative airway use or airway manipulations required during maintenance of anaesthesia. Six patients had sore throat 24 h after procedures and there was no dysphagia or hoarseness.

**Conclusion:**

This pilot study of the LMA protector shows that the device is easily inserted with fast insertion time, providing a reliable and adequate airway seal.

**Trial registration:**

Clinicaltrials.gov Registration NCT02531256. Retrospectively registered on August 21, 2015.

## Background

Airway management is a crucial part of general anaesthesia. The LMA Protector™ is the latest innovation from the inventors of laryngeal mask (Teleflex Medical, Co. Westmeath, Ireland). It is a CE marked single use supraglottic airway device (SAD). It is made primarily of silicone, latex free and allows insertion without the need for digital or introducer tool guidance. Similar to the LMA Supreme and LMA Proseal (p-LMA), the LMA Protector™ has an integral bite-block that reduces the potential for tube damage and obstruction by biting. The device has a fixation system which prevents proximal displacement during use ensuring that the distal end seals around the upper oesophageal sphincter.

**Fig. 1:**
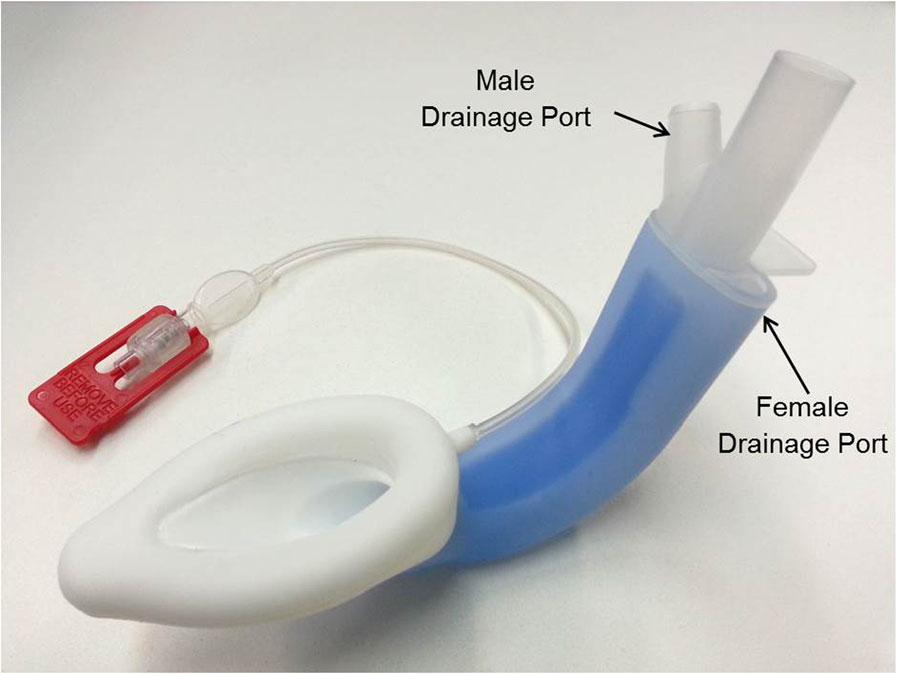
The LMA Protector™ contains two separate drain channels

The features of the LMA Protector™ include a functional separation of the respiratory and digestive tracts. The anatomically shaped airway tube is elliptical in cross section and ends distally at the laryngeal mask. Different from other SADs, the LMA Protector™ contains two drain channels which emerge as separate ports proximally. The drain channels continue distally and enters a chamber located behind the cuff bowl. The chamber further narrows distally into an orifice located at the end of the cuff to communicate distally with the upper oesophageal sphincter. A suction tube may be attached to the male drainage port around the laryngeal region or a well lubricated gastric tube may be passed through the female drainage port to the stomach (Fig. 1).

Although the physical characteristics of LMA-Protector were reported, currently there are limited reports on the role of LMA Protector™ in airway management [1]. We conducted this preliminary assessment in the anaesthetised women who underwent minor gynaecological procedures with spontaneous ventilation in order to evaluate its performance as part of the initial investigation of the airway device by the sponsor.

## Methods

The Singhealth Centralised Institutional Review Board (CIRB Reference: 2013/709/D) approval and clinical trials registration (NCT02531256) was obtained for this study and every subject gave written informed consent. The study period was between October 2014 and January 2015. The inclusion criteria were female patients who were American Society of Anesthesiologists (ASA) class 1 and 2, 21 to 70 years old and were expected to undergo minor gynaecological procedure with general anaesthesia and airway management using a SAD. Patients were excluded if they had a body mass index of 30 kg/m^2^ or more, known gastro-oesophageal reflux, increased risk of aspiration, upper airway pathology and mouth opening of less than 2 cm.

All anaesthetists participating in the study were experienced in the use of LMA Supreme and p-LMA. Each investigator had performed more than 500 LMA insertions. Before recruiting subjects in this study, each anaesthetist was instructed in the use of the LMA Protector using an airway mannikin and used the device to manage the airways of 14 patients. Data from these patients are not included in this analysis.

The Samsoon and Young modification of the Mallampati classification was used to assess the airways of all subjects [2]. We applied standardised monitoring (gas analyser, pulse oximeter, intermittent non-invasive blood pressure monitor, electrocardiogram) in all patients prior to induction of anaesthesia. The LMA Protector cuff was completely deflated and a water-based lubricant was applied to the posterior part of cuff and airway tube. The LMA protector size 3 was utilised for all subjects. After pre-oxygenation, we induced anaesthesia with fentanyl 1.5 to 2 mcg/kg, propofol 2 to 3 mg/kg and maintained anaesthesia with sevoflurane with an end tidal concentration of 2 to 3% in oxygen until the jaw was considered relaxed at the discretion of the investigators during airway manipulation. No neuromuscular blockade was used.

All subjects were placed in a semi-sniffing position. Under direct vision, the tip of the device was pressed flat against the hard palate. Keeping the airway tube close to the chin, the device was rotated inwards in one smooth circular movement similar to insertion of the LMA Supreme, until definite resistance was felt. Ease of device insertion was recorded as ‘no or minimal resistance’, ‘moderate resistance’, ‘severe resistance’ and ‘impossible to pass without excessive force’. We allowed up to 3 attempts at LMA Protector insertion. The number of attempts at insertion and the time to achieve airway (time from pickup of the LMA Protector to the presence of CO_2_ trace on capnography) were recorded. The cuff was then inflated initially with 15mls of air and further inflated to achieve an intra-cuff pressure of 60cmH_2_O using a separate pressure measurement device. The LMA Protector was secured to the patient’s face by adhesive tape.

The anatomical airway position of the LMA Protector was assessed by fiberoptic examination via the airway channel. The view via the airway was scored as follows: Grade 1, clear view of the vocal cords; Grade 2, view of the arytenoids only; Grade 3, view of the epiglottis only; Grade 4, no laryngeal structures visible [3]. Subsequently, fiberoptic examination was also done via the gastric tube to assess the gastric channel patency. The suprasternal notch test was performed by applying a bolus of gel on the male drain port and occluding the female drain port, whilst another investigator applied suprasternal notch pressure.

Suction was then applied to the male port and the presence of negative suction was assessed by feeling for negative pressure at the female port. This is to assess for seal between the gastric access channel, male and female ports. A well lubricated 14 F gastric tube was then passed through the female port into the gastric access channel (Fig. 1). Successful insertion into the stomach was confirmed by auscultation over the stomach during injection of 10 ml air into the gastric tube. We adjusted the cuff pressure to 60cmH_2_O and measured the oropharyngeal leak pressure at this intra-cuff pressure with a fresh gas flow of 4 L/min of oxygen. We closed the expiratory valve of the circle anaesthetic breathing system and noted the airway pressure in the breathing system at which there was equilibration or up to a maximum pressure of 40cmH_2_O.

Failed insertion was defined by any of the following criteria: (1) failed passage into the pharynx; (2) malposition (air leaks, negative tap test results, and failed gastric tube insertion if pharyngeal placement was successful); and (3) ineffective ventilation (maximum expired tidal volume < 8 ml/kg or end-tidal carbon dioxide > 45 mmHg if correctly positioned) [4]. During maintenance of anaesthesia, the number of airway manipulations during procedure (additional cuff inflation, position corrections) and airway complications (intermittent obstruction, complete obstruction, airway leak, need for alternate airway device) were recorded. The subjects were maintained on spontaneous or assisted ventilation throughout the minor gynaecological procedures without neuromuscular blockade.

On emergence from anaesthesia, the LMA Protector was removed by recovery staff when the patient was able to open their mouth to command. During emergence and removal, airway complications and the presence or absence of blood or excessive secretions were recorded. On day 1 postoperatively, subjects were interviewed on the presence or absence of sore throat, dysphagia and hoarseness of voice.

Data were analysed by using SAS (Version 9.4, North Carolina, USA). We used means and standard deviation to describe continuous data; median, interquartile ranges (IQR) and ranges for non-parametric data; and percentages for categorical data.

## Results

We studied 26 females undergoing minor gynaecological procedures, the baseline and demographic characteristics found in Table 1. All subjects were placed in the lithotomy position. The LMA protector size 3 was used in all subjects as provided.

**Table 1:** Baseline and demographic characteristics of subjects recruited

Parametres	Number of patients	Mean (SD)/Median [Range]/ Percentage
Age; years	26	43.3 (11.5)
Body Weight; kg	26	60.5 (9.4)
Body Mass Index; kg/m^2^	26	24.9 (3.4)
ASA Status:		
Grade 1	16	61.5%
Grade 2	10	38.5%
Modified Mallampati Classification		
1	0	0%
2	26	100%
3	0	0%
4	0	0%
Duration of Procedures; min	26	19 [12-32]

The data are represented as mean (SD), median [range] or number (% percentage)

The airway and ventilation profiles for the subjects are reported in Table 2. Overall, the LMA protector was able to maintain good seal and adequate ventilation in all the subjects. Insertion of the LMA protector with subsequent ventilation was possible in all 26 subjects. Successful insertion after the first attempt was achieved in 23 subjects (88.5%) and after the second attempt in 3 subjects (11.5%). There was ‘no or minimal resistance’ to insertion in 25 subjects and ‘moderate resistance’ in 1 subject. The median time to achieve airway was 19.0 s (IQR 17.0 to 21 s, range 14.0 to 58.0 s). The median volume of air to achieve intra-cuff pressure of 60 cmH_2_O was 15 ml (IQR 15 to 17 ml, range 13 to 18 ml). The suprasternal notch test was positive in all 26 subjects.

**Table 2:** Airway and ventilation profile

Successful insertion LMA, *n* (%)	
1st attempt;	23 (88.5%)
2nd attempt;	3 (11.5%)
Resistance on insertion of airway, *n* (%)	
No or minimal;	25 (96.2)
Moderate;	1 (3.8)
Time to successful airway placement; sec	19 (17-21 [14-58])
Volume of air to achieve intra-cuff pressure of 60 cm H_2_O; ml	15 (15-17 [13-18])
Vocal cord visibility under fibre-optic inspection, *n* (%);	
Grade 1;	26(100%)
Grade 2;	0 (0%)
Grade 3;	0 (0%)
Successful gastric tube insertion, *n* (%)	
1st attempt;	24 (92.4%)
2nd attempt;	1 (3.8%)
Not possible;	1 (3.8%)
Suprasternal notch test, *n* (%)	
Positive;	26 (100%)
Negative;	0 (0%)
Oropharyngeal leak pressure; cm H_2_O	25.2 (23-29 [21-30])

The data are represented as number (% percentage) or median (IQR [range])

Fiberoptic inspection through the airway tube was performed in all subjects and the vocal cords were visible in all 26 subjects. Negative pressure suction was felt at the female port for all subjects when suction was applied at the male port. Passage of a gastric tube via the female port was attempted in all subjects. This was successful at the first attempt in 24 subjects, at the second attempt in 1 subject and was not possible in 1 subject. The suprasternal notch test was positive in all subjects. The median oropharyngeal leak pressure was 25.5 cmH_2_O (IQR 23.0 to 29.0 cmH_2_O, range 21.0 to 30.0 cmH_2_O).

The LMA Protector was used throughout the maintenance of anaesthesia for all subjects. There was no airway manipulation or airway complications observed. There were no blood traces or excessive airway secretions observed during emergence in the recovery room. The complications and side effects profile is found in Table 3. At 1 day after surgery, 6 subjects (23.1%) reported sore throat during the interview. There were no cases of dysphagia or hoarseness of voice recorded.

**Table 3:** Side effects associated with the LMA protector

Blood or excessive airway secretion on airway device; %	0 (0%)
Sore throat after 1 day surgery; %	6 (23.1%)
Dysphagia or hoarseness of voice; %	0 (0%)

The data are represented as number (%)

## Discussion

Our initial experience found a high first attempt and overall success rate of LMA Protector insertion and ventilation in this case series of 26 subjects with normal airways. The anatomical position of the airway was optimal in all subjects and most subjects had successful gastric tube insertion. There was no visible blood trace upon removal and only a minority had sore throat after their procedures.

The LMA Protector was designed as a single use device combining the features of earlier laryngeal mask airways to enable ease of insertion, fixation and gastric access. The additional features of the silicone material aim to make the airway tube less rigid and softer, and to reduce mucosal trauma with potential to be used in longer duration procedures. Furthermore, the gastric access allows for larger flow of regurgitants at low pressure to prevent or reduce gastric regurgitations.

The insertion of the LMA protector was easy in all the cases in this pilot study. The first time insertion success rate was 88.5% and the overall success rate was 100%. First insertion success rate was higher for the LMA Supreme than for the p-LMA during spontaneous respiration [3, 5]. Two previous studies of the LMA Supreme reported 92% [6] and 100% [7] first time insertion success rate. Similar to other studies, our study did not report any failures. These features are similar to the LMA Classic (c-LMA), p-LMA and I Gel [8–10].

The LMA protector provides adequate rapid securing of the airway with a median time of 19 s. This is similar to other SADs such as the LMA Supreme, p-LMA, c-LMA and I Gel [10–15]. The LMA protector is a new airway device and the investigators involved had only performed 14 insertions before commencing on the study. With more experience with the LMA protector, first time insertion rates and insertion times could be improved.

The median oropharyngeal leak pressure was 25.5 cmH_2_O. This is similar to some SAD reports such as LMA Supreme and I Gel [10, 12]. The LMA protector also confers drainage of gastric contents compared to the first generation c-LMA [11] However, higher oropharyngeal leak pressures. Van Zundert et al. reported that LMA Supreme oropharyngeal leak pressure was 37 cmH_2_O which was higher than ours [7]. The leak pressure of the LMA Proseal was reported to be 32 cmH_2_O, higher than that of the LMA protector [9, 11]. The lower oropharyngeal leak pressure with the LMA protector should be investigated further, as this may have implications to the maximum inspiratory ventilating pressure and gastric aspiration risk. The gastric tube insertion was successful on a majority of cases (25 out of 26). Most studied SADs also achieved high successful insertion rate of the gastric tube [9, 10, 16].

The presence of double drainage channels, a unique feature of the LMA protector, may reduce the risk of aspiration and regurgitation. The p-LMA has been investigated in a cadaveric study and found to offer significant protection against aspiration [17]. Trivial regurgitation was reported with the use of LMA Supreme, although this was rare [10]. Our present study assessed the seal between the gastric access channel, male and female port to suggest ability for suctioning at the laryngeal region and was found to be present in all subjects. Further studies on the LMA protector would need to be performed in the setting of longer duration of surgery and with the use of muscle relaxation.

The limitations of this study would include the small number of subjects and the short duration of gynaecological procedures. This preliminary study is part of the registration requirements for the sponsor of the LMA protector. The effect of change to silicone material would require longer surgical procedures to manifest, including a reduction in airway complications such as sore throat and hoarseness of voice. Furthermore, the presence of the drainage channel opening was not fully evaluated due to the short duration of procedure as airway secretions usually accumulate over longer periods of time. Only size 3 LMA Protector was utilised in this study, as only this size was provided by the sponsor company. However, optimal positioning of the airway device was obtained in all subjects even if they were not in the recommended weight range for the LMA size. Furthermore, the exact position of the LMA with fibreoptic scope examination is still limited, as malpositions may not be detected adequately [18]. Further studies should be conducted with larger number of subjects [19, 20].

## Conclusion

Overall, the initial experience with the LMA protector has found that this new SAD has a high first attempt and overall insertion success rate. The anaesthetists could rapidly achieve effective ventilation with reliable airway seal. Larger studies with larger number of subjects are needed to investigate the effectiveness of the double drainage channels in the setting of prolonged surgical procedures and with the use of muscle relaxation. The effect of the softer silicone material on postoperative airway complications should also be investigated.

## Abbreviations

ASA: American Society of Anesthesiologists
CIRB: Centralised Institutional Review Board
IQR: Interquartile ranges
SAD: Supraglottic airway device
